# Quitting thresholds in visual search are impacted by target present detection times but not their variability

**DOI:** 10.3758/s13414-022-02591-3

**Published:** 2022-10-18

**Authors:** Mark W. Becker, Andrew Rodriguez, Dana Pontious

**Affiliations:** Department of Psychology, Michigan State University, 316 Physic Rd, East Lansing, MI 48823 USA

**Keywords:** Visual search, Eye movements and visual attention, Change blindness

## Abstract

Models of visual search posit that target absent responses occur when the quitting threshold for the trial is reached before a target is detected, and that feedback about missed targets allows the quitting threshold to be adaptively set to the difficulty of the search task. While these models may effectively capture processes in lab-based tasks, in real-world searches feedback is often impossible to provide. Instead, observers have little information about their errors, and may only be aware of when they successfully detect the target. We posit that in the absence of feedback the time required to find a target might influence quitting thresholds. In three experiments, we investigate how manipulating the mean time and the standard deviation of time to detect a target influence quitting thresholds in target absent trials. To vary target detection times while holding the search stimuli constant, we used an eye-movement contingent change to surreptitiously introduce a target near fixation at a particular time. Results show that decreasing the mean time to find a target also decreases the number of items inspected and reaction time in target absent trials, the hallmark of a shift in the quitting threshold. By contrast, varying the standard deviation around a fixed mean had no impact on target absent search times. These findings suggest that people are sensitive to the typical time required to find a target in a given task and use that information to flexibly adjust target absent quitting thresholds, but people are not sensitive to the variability.

## Introduction

While there has been a great deal of visual search research investigating how people detect the presence of targets, the processes involved in making a target absent response have received far less investigation. Initially, the reported target absent-to-present search slope ratios of 2:1 were taken as evidence that search is exhaustive for target absent trials but is self-terminating for target present trials (Treisman & Gelade, 1980). However, subsequent work has challenged the robustness of the 2:1 search slope ratio, particularly at small set sizes (Pashler, 1987; Wolfe, 1998), and has challenged whether target absent search is exhaustive (Snodgrass, 1972; Zandt & Van Zandt & Townsend, 1993), raising questions about how search trials are terminated with a target absent response. The most thorough model of these target absence responses was posited by Chun and Wolfe (Chun & Wolfe, 1996) and integrated into Wolfe’s Guided Search model (Wolfe, 2021) starting with its second version (Wolfe, 1994).

Under this model there are two-stages of visual search, an initial parallel analysis of the search array results in a priority map in which each item’s activation is based, at least in part, on its similarity to the search target. A second focal attention stage then inspects items serially starting with the item with the highest activation. If that item is determined to be a distractor, the item with next highest activation is attended and evaluated. This process continues until either a target is detected or a quitting threshold is reached. This quitting threshold is inversely related to an activation threshold of the priority map – items with high enough activation are included in the second serial inspection stage while those that do not reach the activation threshold are not. Thus, the activation threshold determines the number of items that must be inspected before the participant makes a target absent response, and thereby dictates the amount of time that will be spent before making a target absent response – the quitting threshold (Wolfe & van Wert, 2010). This method of thresholding allows for efficiency because it allows a participant to terminate a trial with a target absent response without performing an exhaustive search – the participant needs not inspect items that are unlikely, based on their activation, to be a target.

The Chun and Wolfe (1996) model provides for further efficiency in this process by allowing the quitting threshold to be set flexibly during a particular search task. Their model of this process is akin to a staircase method. When a participant makes a successful target absent response, the activation threshold is increased, thereby resulting in fewer items being above the threshold, and thus inspected, prior to making a target absent response in the subsequent trial. If the threshold gets too high, the subject will become likely to miss a target. When a miss occurs, the activation threshold is reduced for the subsequent trial. This process of increasing the threshold following correct target absent responses and decreasing the threshold following misses allows search to become more efficient and tuned to the difficulty of the search task.

The model is supported by trial-by-trial reaction time (RT) patterns, which show a saw-tooth pattern of gradual speeding of target absent responses following successful target absent responses, and dramatic slowing following a miss (Chun & Wolfe, 1996). The model may also be able to account for the low prevalence search effect, the finding that as targets become rare, target absent RTs become faster and misses increase (Godwin et al., 2014; Mitroff & Biggs, 2014; Peltier & Becker, 2017; Wolfe et al., 2007); with few targets, there are frequent correct target absent responses leading to dramatic increases in the activation threshold, thereby producing fast target absent responses and high miss rates, the hallmark of a low quitting threshold (Wolfe & van Wert, 2010).

Finally, the Chun and Wolfe model provides for a second factor that can influence search performance, the decision criterion that is used when doing the serial evaluation of items to determine whether the currently inspected item is a target or not. In theory, this decision criterion can also be manipulated. Work investigating how target prevalence rates influence visual search suggest that this criterion can be modelled using a drift diffusion decision model, and thus fixation dwell times can be used to indicate a shift in the decision boundary/criterion (Peltier & Becker, 2016). For example, if the decision criterion for identifying an item as a distractor becomes more liberal, the amount of information required to reach the distractor decision boundary will decrease and, assuming that information accumulation/drift rate is constant, the amount of time required to make that evaluation will decrease thereby reducing dwell times on distractors.

Despite the strength of this model in explaining lab-based search tasks, the model has some severe limitations. Most notably, the entire adaptive process depends critically on trial-by-trial feedback, and in particular feedback about misses (Chun & Wolfe, 1996). While many lab-based search tasks involve this type of feedback, most real-world searches do not. As a result, the model is limited in its ability to explain search processes beyond the lab. Indeed, if one considers real-world search contexts, like looking for scissors in the junk drawer, TSA agents searching baggage, or a radiologist searching scans for signs of disease, feedback is rare and feedback about misses is essentially non-existent. For instance, finding the scissors in the draw provides some knowledge that there was a successful hit (you have the scissors), but failing to find them provides no information about a miss (the assumption is that the scissors are not in the drawer – not that they were present but missed). In short, in the absence of trial-by-trial feedback, there may be information about the time it takes to find the target, but there is no information about whether a miss occurred.

Returning to the Chun and Wolfe model, the failure to have information about misses eliminates the mechanism that allows quitting thresholds to be adaptively set to the specific search task. In addition, the model provides no mechanisms for information about the time that it takes to find a target, the information that may be present in the absence of trial-by-trial feedback, to influence quitting thresholds. We posit that search quitting thresholds are likely to be adaptively set even in the absence of trial-by-trial feedback and that the typical time that is required to find a target, when present, is a likely factor influencing this adaptivity.

However, designing an experiment to empirically evaluate how the time required to find a target influences quitting thresholds presents some unique challenges. Typically, a major factor determining the average time required to find a target is the amount of pre-attentive guidance the task affords. This guidance is a function of several factors (see Wolfe, 2021) including the target’s bottom-up saliency (Itti et al., 1998), history effects (Awh et al., 2012), scene semantics (Torralba et al., 2006), and the ability to guide search in a top-down manner to the target. Changes in these guidance factors can influence the level of activation of items within the priority map. Thus, even with a fixed quitting threshold, the number of items that reach the threshold (and thus the time required before making a target absent response) can be influenced by changes in these guidance factors. So, to avoid potential confounds and isolate the influence of target present RTs on quitting thresholds requires the ability to manipulate the RT for hits, without influencing these guidance factors. In other words, what is required is the ability to manipulate target present RTs while holding the visual characteristics of the search task constant.

To accomplish this, we utilized an eye-movement contingent change to a search array that allowed us to introduce the target, near fixation, after a prespecified amount of time. That is, the target was introduced during the first saccade after the prespecified time. These types of eye-movement contingent changes typically go unnoticed (Henderson & Hollingworth, 2003; McConkie & Zola, 1979), allowing us to surreptitiously insert the target, thereby influencing target detection times while holding the visual characteristics of the arrays constant. In two experiments (Experiments 1 and 2) we systematically varied the mean amount of time that elapsed before introducing the target. These two experiments were identical except for the target prevalence rate (50% and 80%), allowing us to determine whether additional evidence of the mean time to find a target (via more frequent trials) increased the influence that target present RTs had on quitting thresholds. In a third experiment, we held the mean time to find a target constant, but varied the standard deviation of the distribution of target present detection times around this mean, to investigate whether people were sensitive to the variability in search times and whether that variability influenced quitting thresholds. For comparison, we also ran a Control Experiment involving standard search methods (i.e., the target was present from the beginning of target present trials) with the same stimuli in the experiments. To preview our results, we find that the mean target present search times influence quitting thresholds, but changes in the variability do not influence quitting thresholds.

## Methods

### Participants

All participants were undergraduates from Michigan State University who reported normal or corrected-to-normal vision and participated for course credit. Participants gave written informed consent under the study protocol approved by the Institutional Review Board. The Control Experiment had 20 participants. Experiment 1 had 39 participants, one of whom had technical difficulties with the eye-tracker, leading to data from 38 participants (15 male, 23 female). This sample size was selected to achieve power of .85 to find an effect size of .5 in a paired-sample t-test (Faul et al., 2007).

### Displays and eye movement contingent changes

**Fig. 1:**
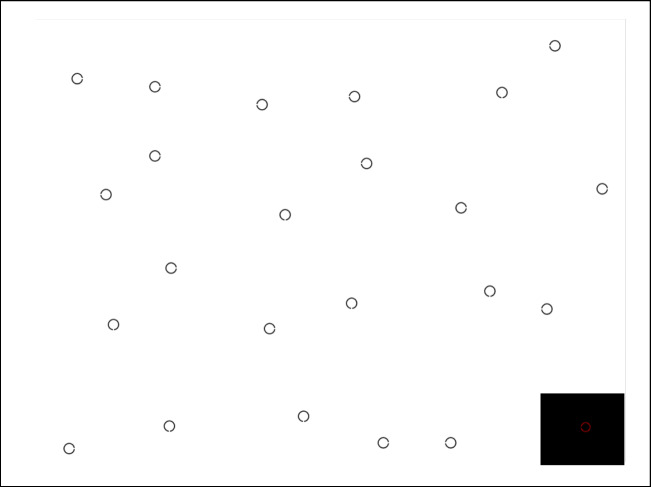
A mock version of a distractor search array. In this version the Cs are slightly larger and printed in black on a white background for easy visualization. In the real experiment the Landolt Cs were dim red on a black background making them more difficult to see. One example of a stimulus on black is present in the lower right of the array

Displays were presented on a 24-in. Dell monitor with a 16:9 aspect ratio. Each display (Fig. 1) was created by segmenting the screen into a 6 x 4 grid of regions and placing a small Landolt C in each region, with random jitter within the region, with the caveat that the item could be no closer than 1.5° from the edge of the region, thereby insuring at least 3° between all Cs. The Cs were dark red on a black background, and had a diameter of .8°, line widths of .08°, and small breaks (.1°) that could appear at one of the cardinal directions. The combination of small, low-contrast stimuli and small breaks were designed to ensure that identification of the break required fixation and helped ensure that our surreptitious, eye-movement-contingent changes were not detected. Each trial began with a drift correct for the eye tracker and eye movements were monitored via an EyeLink 1000 eye tracker throughout the search trials. The task was to search for a C with a break on the top and make a present/absent response via a button press. There was no feedback provided in any of the experiments.

The control experiment consisted of a standard search paradigm with targets present on 50% of trials, and in target present trials the target was presented from the beginning of the trial. Data from one subject were eliminated due to extremely high errors. The RTs from target present and target absent trials were calculated for the remaining 19 subjects. These means were 6.17 s (SE = .35 s) and 11.9 s (SE = .71 s), respectively. These values were used to determine the two timers for the target present trials in the real experiments. We chose the timers for target present trials to be 2 and 4 s, because these were faster than the typical target present times, and very few target absent responses were made prior to 4 s.

For Experiment 1, at the beginning of every trial all 24 Cs were distractors that had breaks on the right, left, or bottom. If the trial was a target absent trial, the search proceeded until the subject made a response. If the trial was a target present trial, as soon as the array was presented a timer began. In one block of trials the timer was set to 2 s and in a second block of trials the timer was set to 4. After the timer expired, an eye-movement velocity trigger (which fired if the velocity of the eye reached 30°/s, or the acceleration reached 8000°/s) was activated so that during the next saccade, all items on the screen would be replaced with targets that had a break on the top. This replacement was performed during the eye movement, rendering the change invisible to the subjects, which was verified with a post-experiment question. We changed every item to be a target to ensure that the item at the end of the saccade would be a target, regardless of where the saccade was directed. Pilot work found that people often make a saccade away from the target and then saccade back to the target, consistent with the suggestion that saccades might leave an item prior to the completion of processing the fixated item thereby requiring refixation (Henderson, 1992). Thus, if the participant made an additional saccade, a second velocity trigger changed all of the items except for the one nearest to prior fixation back to the original distractor at that location. At the completion of this second change, the display consisted of 23 distractors and one target at the location of the last fixation.

### Procedure

After participants filled out informed consent, they used a chin rest while the eye-tracker was calibrated, using the EyeLink 1000s standard 9 location calibration routine. After successful calibration, the program showed written instructions that included examples of the target and distractor stimuli. After the participants finished reading the instructions the experiment began. There were two blocks of trials, with different timers (2 and 4 s) for the target present trials. The order of blocks was counterbalanced across participants. Each block began with 14 practice trials to familiarize participants with the task and the typical amount of time it took to find a target. Practice trials were followed by 60 trials in each block, for a total of 28 practice and 120 real trials. Targets were present in 50% of the trials. If the trial was supposed to be a target present trial and the participant responded before the target was displayed (e.g., before the timer elapsed), the trial was removed from analysis. This occurred rarely, accounting for an average of 2.12% of trials in Experiment 1. There was a break for a rest between the two blocks of trials.

## Results

While our main analyses of interest involve the RT data, for completeness we analyzed accuracy. False alarms were fairly low for both the 2-s (M = 6.9%; SE = 2.5%) and the 4-s condition (M = 5.6%; SE = 2.6%), and did not differ by condition, t(37) = 1.21, *p* = .24. The hit rates were somewhat low for both the 2-s (M = 67.7%; SE = .3.3%) and the 4-s (M = 70.6%; SE = 3.2%) conditions, and did not differ significantly from one another, t(37) = 1.40, *p* = .17. The low hit rates are somewhat surprising given that the target appeared at fixation. However, we note that the target was made particularly difficult to identify so that eye movements were required and to make it unlikely that people would detect the change in the display. Further, these low hit rates did not significantly differ (2-s: t(55) = 1.85, *p* = .07; 4-s: t(55) = 1.36, *p* = .18) from the hit rates in the control condition (M = 78.0%, SE = 4.2%), suggesting that the low hit rates were not a direct result of our eye-movement-contingent manipulation.

**Fig. 2:**
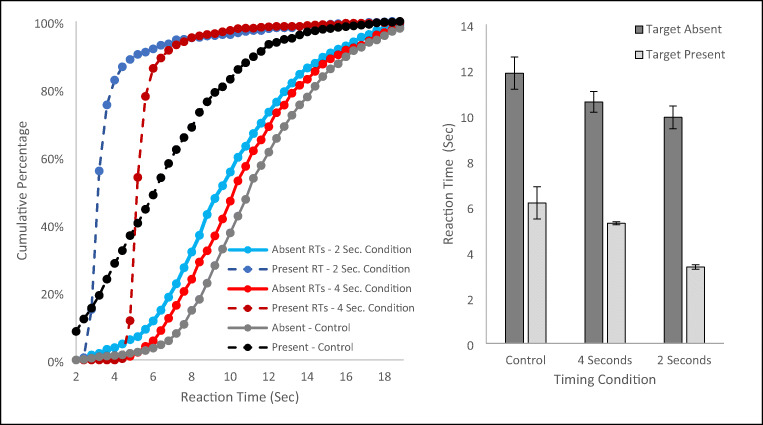
The left panel plots the cumulative percentage of reaction times (RTs) for each condition of Experiment 1. The right panel plots the mean of subjects’ median RTs for each condition. Each panel also presents data from the Control Experiment for comparison. Error bars are the standard error of the mean

To visualize the RT data we created histograms of the RTs for trials in each condition and used them to calculate the cumulative percentage of RTs for the condition (see Fig. 2 left panel). For statistical analyses, we calculated each subject’s median RT for target present and target absent RT trials for each timer condition block and performed our analyses on these medians (see Fig. 2 right panel). While the figure presents the data from the Control Experiment for comparison purposes, to begin our analyses we performed an omnibus 2 (present/absent) x 2 (2-s timer/4-s timer) repeated-measures ANOVA for the conditions in Experiment 1. This analysis found a significant main effect of present/absent, F(1,37) = 130.3, *p* < .001, partial eta squared = .779, a main effect of the timer condition, F(1, 37) = 46.7, *p* < .001, partial eta squared = .558, and a significant interaction, F(1, 37) = 12.7, *p* = .001, partial eta squared = .255. The source of the interaction was that increasing the timer from 2 to 4 s resulted in sizable increase in target present RTs (mean increase = 1849.6 ms, SE = 95.87), but the increase in the target absent RT was much smaller (mean increase = 652.15 ms, SE = 339.48).

### Target present trials

The target present data in Fig. 2 clearly show that the manipulation was successful; the RT for the 4-s condition was about 2 s longer than the RT for the 2-s condition. The difference between these two conditions was confirmed by a paired t-test, t(37) = 19.84, *p* < .001, d = 3.22, and a single-sample t-test on difference scores between the two conditions found that the mean difference of 1.85 s (SE = .095 s), did not differ significantly from 2 s, t(37) = 1.33, *p* = .13.

It is also worth noting that RTs for hits in both target present conditions of Experiment 1 were faster than the RTs for the Control Experiment (a standard search in which the target was present from the beginning of the trial). These differences were confirmed with a one-way ANOVA with three levels, F(2, 92) = 76.56, *p* < .001, partial eta squared = .625, and Bonferroni-corrected post hoc analyses confirmed that both conditions in Experiment 1 were significantly faster than the target present responses in the control, both *p* < .002. Finally, it is worth noting that there was little variability in target present RTs in Experiment 1 when compared to Control Experiment.

Finally, we compared the RTs for the trials where the target was missed with the RTs for the correct target absent responses to make sure these were not radically different from one another. For the 2-s condition, the miss RTs (M = 9.79 s, SEM = .59) were marginally slower, t(34) = 1.97, *p* = .056, from the correct target absent responses (M = 9.40 s, SEM = .55). For the 4-s condition, the miss RTs (M = 11.03 s, SEM = .57) were significantly slower, t(34) = 2.73, *p* = .007, than the target absent RTs (M = 10.3 s, SEM = .49). In theory, the miss responses should be similar to the correct target absent responses, so it is somewhat surprising that the miss RTs are longer. However, we are not overly concerned about this finding since it did not replicate in Experiment 2 (see below), a number of subjects made very few misses making raising questions about the reliability of these miss RT data, and it is possible that the miss trials are trials where participants are somewhat off-task, which might lead to longer RTs.

In sum, these target present analyses suggest that our manipulation was successful in altering the time to detect the target in target present trials.

### Target absent trials

The critical comparison was between the target absent RTs for the two timer conditions in Experiment 1. A paired t-test revealed a significant difference in target absent RTs, t(37) = 2.13, *p* = .04, d = .345, with longer RTs in the 4- than the 2-s condition. However, the magnitude of the difference in RTs between conditions was far smaller (M = .652 s, SE = .339) than the roughly 2-s difference in target present RTs, t(37) = 3.56, *p* = .001, d = 5.78. We also compared the target absent RTs in each of the timer conditions in Experiment 1 to the target absent RTs from the Control Experiment (which had longer target present RTs than either block in the current experiment). An independent-sample t-test revealed that RTs for the 2-s block were faster than target absent RTs for the Control, t(55) = 2.59, *p* = .012 d = .727, but the 4-s block was only marginally faster than the Control, t(55) = 1.95, *p* = .057, d = .547.

## Discussion

The results from Experiment 1 clearly show that decreasing the amount of time required to find a target when present also impacts the amount of time that a person searches prior to making a target absent response. However, that finding on its own does not clearly indicate the search mechanism that results in this shift in search time. In theory, the reduction in search time could be caused by a reduction in the number of items inspected prior to responding target absent, the hallmark of a reduction in the trial-wide quitting threshold. The reduction in RTs could also be caused by decreasing the time required to evaluate whether each item is a target or not during the serial inspection stage, and indication of a shift in the decision criterion. Of course, these two processes are not mutually exclusive – it is possible that both are impacted by target present search times.

The eye-movement data provide a method to evaluate whether target present detection times influence these two putative mechanisms. An analysis of the percentage of array items that were fixated prior to making a target absent response revealed that fewer items were inspected in the 2-s (M = 77.6%; SE = 3.0%) than in the 4-s timer condition (M = 80.4%; SE = 2.6%). While this difference was only marginally significant, t(37) = 2.00, *p* = .053, it suggests that target present detection times influence the quitting threshold.

To evaluate possible changes in the decision criterion, we compared average dwell times on fixated distractors for the target absent trials as a function of timer condition. Dwell times did not differ significantly, t(37)=.29, *p* = .77, between the 2-s (M = 346.95 ms, SE = 11.71 ms) and 4-s (M = 348.98 ms, SE = 11.69 ms) timer conditions. Similarly, we compared the dwell time on successfully detected targets and again found no significant difference, t(37) = .32, *p* = .75, between the 2-s (M = 674.28 ms, SE = 36.41 ms) and 4-s (M = 683.58 ms, SE = 31.99 ms) timer conditions. The dwell-time findings suggest that the target present RT has little effect on the amount of time spent scrutinizing each item during search and thus probably has little impact on the item-by-item decision criterion.

Thus, while decreasing the mean time to find a target in target present trials did result in a decrease in the quitting threshold, the impact on target absent RTs was somewhat small relative to the 2-s change in target present detection times. In addition, part of this small effect may have been due to the somewhat high miss rates, which limits the number of trials where people experience the modified target present search times. In an attempt to address both of these issues, in Experiment 2 we replicate Experiment 1 while increasing the proportion of target present trials.

## Experiment 2

The methods of Experiment 2 were identical to Experiment 1 with one change. In this experiment we had 80% of the trials as target present trials. We did this with the belief that providing more evidence about the typical time that it takes to find a target, when present, may have a more robust impact on the quitting thresholds for target absent trials. A new set of 37 participants (12 male, 25 female) completed Experiment 2.

### Results

Hits were moderately high (2 s: M = 69.92%, SEM = 2.58%; 4 s: M = 69.35%, SEM = 2.69%) and did not differ significantly by condition, t(36) = .23, *p* = .82 or from the hit rate (M = 78.0%, SEM = 4.2%) in the control experiment, both t(54) < 1.8, both *p* > .08. False alarms were fairly low (2 s: M = 2.93%; SEM = 1.03%; 4 s: M = 3.60%; SEM = 1.27%) and also did not differ by timer condition, t(36) = .44, *p* = .66. The RTs for the trials where subjects missed the target (2 s: M = 9.01 s, SEM = .49; 4 s: M = 9.97 s, SE = .51) did not significantly differ from the target absent RTs for either condition (2 s: M = 9.06 s, SEM = .49; 4 s: M = 9.98 s, SEM = .53), both t(36) < .25, both *p* > .8.

**Fig. 3:**
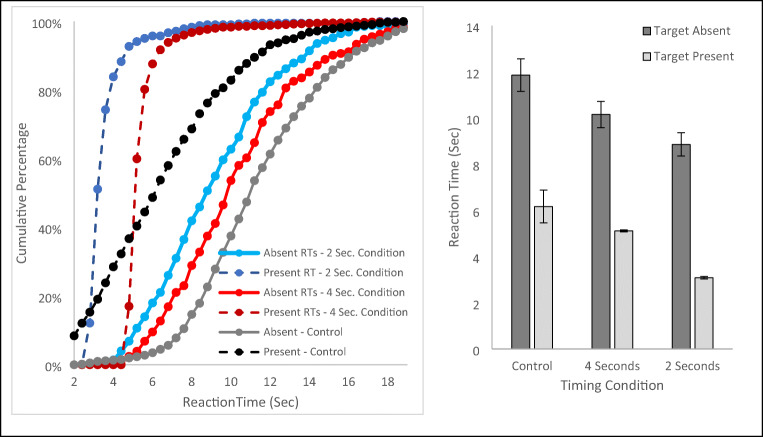
The left panel plots the cumulative percentage of reaction times (RTs) for each condition of Experiment 2. The right panel plots the mean of subjects’ median RTs for each condition. Each panel also presents data from the Control Experiment for comparison. Error bars are the standard error of the mean

To visualize the RT data, we again created histograms of the RTs for trials in each condition and used them to calculate the cumulative percentage of RTs for the condition (see Fig. 3 left panel). Statistical analyses were performed on each subject’s median RT data for target present and target absent RTs for each timer condition (see Fig. 3, right panel).

Like Experiment 1, a manipulation check on the target present RTs revealed a significant effect of the timer manipulation, t(36) = 39.09, *p* < .001, d = 6.43, and the difference in RTs between the two conditions (M = 2.06, SE = .053) was not significantly different from 2 s, t(36) = 1.18, *p* = .248, d = .19. The critical comparison of target absent RTs between the timer conditions again found significantly longer RTs in the 4- than the 2-s timer condition, t(36) = 3.35, *p* = .002, d = .551. While the magnitude of this difference was numerically larger than in Experiment 1 (M = 1.08, SE = .32), again it was far smaller than the roughly 2-s difference in the target present conditions, t(36) = 3.08, *p* = .004, d = .506, and did not differ significantly from the difference in Experiment 1, t(73) = .78, *p* = .44. Finally, we again used independent-samples t-tests to compare the target absent RTs from each of the blocks of this experiment to the Control. The 2-s timer block produced faster target absent RTs than the Control, t(54) = 3.28, *p* = .002, d=.926, but the 4-s timer block did not reach significance, t(54) = 1.90, *p* = .062, d = .537.

In sum, the results of Experiment 2 replicate those of Experiment 1 showing that manipulating target present RTs influences target absent RTs, but increasing the target prevalence rate from 50% to 80% had little impact on the effect that target present RTs had on target absent RTs.

We again used the eye movements to evaluate the extent to which the changes in target absent RTs were due to changes in the quitting thresholds and/or changes in the item-by-item decision criterion. Significantly fewer items of the array items, t(36) = 2.40, *p* = .022, d = .40, were fixated in the 2-s (M = 77.02%, SEM = 2.5%) than the 4-s timer (M = 80.93%, SEM = 2.3%) condition. This finding is consistent with Experiment 1 and is the hallmark of a shift in the trial-wide quitting threshold.

Unlike Experiment 1, in Experiment 2 there was also a difference in the mean dwell times on items in target absent trials. Dwell times were significantly briefer, t(36) = 2.73, *p* = .01, in the 2-s (M= 331.49 ms; SEM = 10.11) than the 4-s (M = 344.56; SEM = 11.25) timer condition. However, like Experiment 1, there was no significant change, t(36) = .46, *p* = .65, in the dwell time on targets between the two-button (M=720.35 ms, SEM = 40.51) and four-button (M=734.12 ms, SEM = 40.15) conditions. Thus, for Experiment 2 it seems that target present RTs influence both the overall trial quitting threshold and the item-by- item decision criterion for distractors but not targets. We address why the evidence for the latter only occurred in Experiment 2 in the *General discussion*.

## Experiment 3

In Experiments 1 and 2 we found evidence that people adjust their quitting thresholds based on the typical time required to find a target when it is present. If it typically takes less time to find a target, the quitting threshold is lowered resulting in faster target absent search times. That finding is consistent with other findings that suggests that people are sensitive to the statistical regularities in their environment and that those statistical regularities can influence visual processes.

Given the above data suggesting people are sensitive to the mean RT, in Experiment 3 we investigated whether people would also be sensitive to the standard deviation around the mean. We hypothesized that one effective approach to setting an appropriate quitting threshold would be to consider both the mean and the standard deviation of the time required to find a target. One potential model for this would be that people should set their quitting threshold equal to the mean time to detect a target plus some “fudge factor.” It would make sense if the magnitude of this fudge factor scaled with the variability in target present detection times; if the time required to find a target varied widely, one might want to have a large fudge factor – or search for a generous time beyond the mean time required to find the target. By contrast, if there was little variability in the standard deviation of target present detection times it might makes sense to have a small fudge factor – to search just a little longer than the typical target takes to find.

To explore this possibility, Experiment 3 was identical to Experiment 2 except that we set the target present timer to have a mean of 3 s and manipulated the variability around that mean. In one condition, on a given trial the timer was drawn from a normal distribution of possible times with a mean of 3 s and a standard deviation of .1 s. In a second condition, on a given trial the timer was drawn from a normal distribution with a mean of 3 s and a standard deviation of 1 s. We also decided to implement this standard deviation manipulation as a between-subjects factor to avoid the possibility of subjects calculating variability across blocks. Given that between subjects experiments typically have less power than within, we also doubled the number of trials in each condition: each participant performed 28 practice trials and then completed 120 trials within a single condition. Like Experiment 2, 80% of trials were target present to give an opportunity for subjects to be exposed to the variability of target present search times. Fifty-six subjects (19 male, 37 female) participated in Experiment 3. In all other respects, the methods were identical to Experiment 2.

### Results

**Fig. 4:**
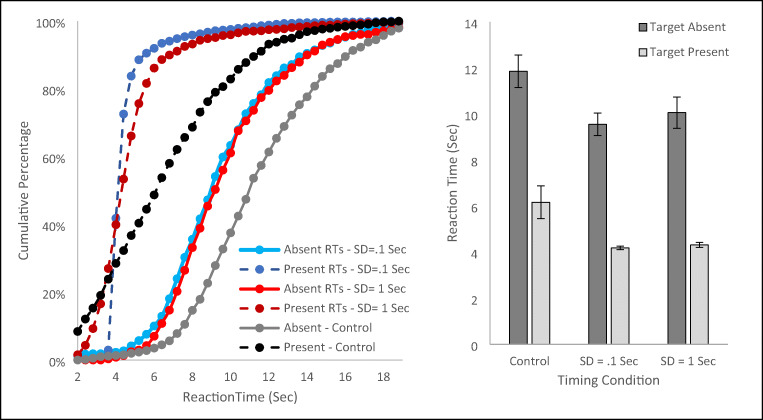
The left panel plots the cumulative percentage of reaction times (RTs) for each condition of Experiment 3. The right panel plots the mean of subjects’ median RTs for each condition. Each panel also presents data from the Control Experiment for comparison. Error bars are the standard error of the mean

We used the same approach as above to generate the cumulative percentage of RTs (see Fig. 4 left panel) and means for each condition (see Fig. 4 right panel). Given that the mean of the target present timers was 3 s in both conditions, we anticipated that the target present RTs should be equivalent in the two conditions. This was verified by an independent samples t-test, t(54) = .301, *p* = .765, d = .081. As a manipulation check, we eliminated RTs over 9 s and calculated the standard deviation within each subject of the hit RTs for the two groups. The standard deviation for target present RTs should be noticeably larger for the group with a 1-s standard deviation for target present timers than the group with a .1-s standard deviation for timers. An independent-samples t-test verified this difference, t(54) = 7.587, *p* < .001, d = 2.033, with a higher mean standard deviation for the group with the more variable timers (M = 1.14 s, SE = .026) than the group with the less variable timers (M =.635 s, SE = .057). For completeness, we performed the same type of analysis on the variability of target absent RTs, although with a more liberal cutoff of 24 s since these RTs tended to be far longer than the target present RTs. The standard deviation of target absent RTs in the .1-s condition (M = 2.14 sec, SEM = .17) were *larger* than the standard deviation in the 1-s condition (M = 1.71 s, SEM = .15), although the difference did not reach significance, t(54) = 1.86, *p* = .07. Thus, our manipulation was effective at increasing the variability of the target present responses, but the manipulation did not seem to impact the variability of target absent responses.

The critical comparison was whether those differences in the variability of target present RTs influenced the target absent RTs. An independent-samples t-test found no difference between the target absent RTs for the two groups, t(54) = .077, *p* = .939, d = .021. Thus, we found no evidence for a larger “fudge factor” being applied to the quitting threshold when target detection times are more variable. However, interpreting a null result via hypothesis testing is a weak statistical decision, so we also performed a Bayes analysis for two independent normal distributions. The Bayes factor was 4.96 for the null hypothesis over the experimental hypothesis, providing moderate evidence in favor of the null hypothesis.

While the main purpose of this experiment was to investigate how variability in target present RTs influence target absent RTs, we also compared the target absent RTs in this experiment to the target absent RTs in the Control Experiment, as a final test of whether differences in mean target present RTs influence the target absent RTs. An independent samples t-test found that the target absent RTs were significantly shorter, 1-s condition: t(43) = 2.50, *p* = .016, d = .754; .1-s condition: t(47) = 2.77, *p* = .008, d = .81, for Experiment 3 than the Control. This pattern provides further evidence that people are sensitive to the mean time to detect a target when present and use that information to adjust target absent quitting thresholds, even when there is substantial variability around the mean time to detect a target.

## General discussion

Using an eye-movement contingent change paradigm we were able to surreptitiously insert a target into similar arrays at various timepoints. This allowed us to examine the influence that the time to detect a target, when present, had on the time that people would search in target absent trials. Our results show that mean time to detect a target influences search times for target absent trials; when the time to detect a target was decreased, there was a significant decrease in the time that subjects searched prior to executing a target absent response. These impacts were not only statistically significant, but they were also quite large – for instance in the 2-s condition of Experiment 2, the target absent RTs were over 3 s, or 25%, faster than in the Control Experiment where the stimuli were presented without our manipulation. These data clearly show that the target present RTs can influence the target RTs.

Further, our analyses of eye movement data provide strong evidence that these changes in target absent RTs are caused by changes in the overall trial quitting thresholds. That is people inspected (fixated) fewer items before executing a target absent response in the 2-s than in the 4-s target present conditions. This pattern of fewer item inspections and faster RTs is the hallmark of a lower quitting threshold.

In Experiment 2, we also found evidence that the target present search times influenced dwell times on distractors, suggesting that the manipulation of target present RTs influenced the time required to identify distractors. One possible interpretation of this change is that with shorter target present RTs there is a shift in the item-by-item identification criterion, biasing the criterion toward identifying items as distractors. However, there was no indication of such a shift in decision criterion in Experiment 1, when targets were less frequent.

Why should speeding target present detection times only influence distractor dwell times when targets are frequent? The reduction in distractor dwell times can be modeled as moving the starting point of the diffusion process toward the distractor detection boundary and away from the target detection boundary. We have previously argued that changes in target prevalence produce such a shift (Peltier & Becker, 2016). However, such a shift in the starting position should produce an increase in target detection times that co-occurs with the reduction in distractor identification times – a pattern we do not see here. A second way to model this in a drift diffusion model is as a shift in the decision boundary for identifying an item as a distractor closer to the original drift starting point, which would reduce distractor identification times without impacting target identification times – the pattern we found in Experiment 2. However, moving the starting point toward the target boundary making such a shift in the decision boundary increases the likelihood of missing targets. People’s willingness to make a shift that speeds search at the risk of increasing misses may depend on their belief that they are performing well. In the absence of feedback, their belief in performing well may be based on how frequently they find targets. When the prevalence rate of targets increases, even with the same detection rate, the overall number of targets found increases and thus people may feel like they are doing well, and therefore may be willing to shift the decision boundary for distractors closer to the drift starting point.

While this explanation is speculative, a similar logic was recently argued in a paper examining how low prevalence impacts the decision criterion in a two-alternative classification task (Lyu et al., 2020). The authors found that, in the absence of feedback, decision criteria became more liberal, expanding the category boundary of the rare target. On the basis of these findings, they argued that, in the absence of feedback, people are sensitive to how frequently they execute the different possible responses. When a given category becomes rare, people notice that they are not identifying many objects as belonging to that category and allow the category to expand, thereby increasing the number of times they execute the response associated with that category.

Following this logic, in the 2-s condition of Experiment 1 participants were making target present responses on only about a third of the overall trials (67% hit rate x 50% prevalence rate), while in Experiment 2 they were making target present responses on more than half of the trials (71% hit rate x 80% prevalence rate). When subjects were responding with relatively few target present responses (Experiment 1), they may have been concerned that they were missing targets and maintained a criterion that required more thorough scrutiny of distractors before determining that they were not targets, even though targets, when found, were found quickly. By contrast, when the targets were found frequently (Experiment 2) the speed with which they were found may have a larger influence on the decision criterion. Another way to think of this is that people are willing to shift to a more liberal criterion when they believe the search to be easy. This evaluation of ease of the search may be based on a combination of both the speed of detection and the absolute number of detections. When overall target detections are high the speed of detections influence the decision criterion. When overall target detections are low, the speed of detection is not as influential as the low overall detection rate.

We also tested whether the variability around a mean target present RT would influence target absent RTs. The motivation for doing so is that we posited that people might set their quitting threshold to the mean of the target present RT plus some fudge factor; and we reasoned that the fudge factor might be related to how variable RTs were for the target present trials. If the RTs for target present trials were tightly grouped, once the trial had progressed beyond that tight grouping one could be fairly confident that the target was absent. By contrast, if the RTs were extremely variable for target present trials, one might have to search for longer before becoming confident that the target was absent. However, our conjecture that the variability of target present RTs would also influence target absent RTs did not hold; people seemed very insensitive to this variability.

While the findings of a lack of sensitivity to variability is somewhat disappointing, it makes the interpretation of the effect of changing the mean easier to interpret. The way we controlled the timing of the detection of the target in Experiments 1 and 2 also decreased the variability in finding those targets – both mean and standard deviation of detection times were altered compared to the control condition. Thus, if both had an influence, parsing out the influence of each may have been challenging. The fact that variability seemed to have no effect avoids this potential pitfall.

In fact, in some regards the data from Experiments 1 and 2 provide additional evidence of how insensitive people were to variability. In those experiments the target was almost always found within a very tight window, yet people searched far beyond that window in target absent trials. For instance, in the 2-s condition of Experiment 2, the mean within subject standard deviation for target present RTs was about .65 s. Even so, subjects in this condition searched for an average of 5.8 s longer in the target absent trials than the target present trials – or almost 9 SDs more than the mean target detection time!

Thus, our data clearly support that, in the absence of feedback, participants are sensitive to the mean target present detection times and use that information to adaptively set the quitting threshold that determines when a target absent response is made. The data are equally clear that, at least with the magnitude of different variabilities we presented, people seem insensitive to the variability in target present RTs.

These findings have implications for existing models of target absent decision making. To review, current models suggest that feedback, and particularly feedback about misses, are the driving mechanism allowing one to adaptively set quitting thresholds (Chun & Wolfe, 1996; Wolfe & van Wert, 2010). Our results suggest that current models should be expanded to include a mechanism by which mean target present search times can impact target quitting thresholds. In addition, in the current experiments the influence of target present RTs did not depend on feedback (none was provided). Thus, this addition is important because it provides a mechanism for adaptively setting quitting thresholds in the absence of feedback, as is common in real-world search tasks.

Finally, we should acknowledge that the current approach was intentionally contrived to provide good experimental control, allowing us to isolate the impact of target present detection times, while holding other factors that could influence search times and quitting thresholds constant. To do so we used a very difficult search task, in which target detection took a long time during a traditional search. While this approach had the benefit of allowing us to significantly speed target present RTs via our manipulation, it did minimize the factors that can help guide search. In the most recent iteration of Wolfe’s Guided Search Model (Wolfe, 2021), there are five factors (e.g., history, reward, scene semantics, top-down and bottom-up guidance) that can influence guidance by influencing the level of an item’s activation within a priority map. In our experiments, almost all of these guidance features were eliminated (with only the possibility of top-down feature guidance remaining but even it would be relatively ineffective with these stimuli). So, one possibility is that in the presence of those guiding factors, the influence of mean target detection time may be relatively minimal and ineffectual.

Those guidance factors can impact the time to find a target. In addition, they can influence quitting times, and thus provide for adaptively setting search, without necessarily requiring a shift in the quitting threshold per se. For instance, suppose there is a fixed quitting threshold that acts on a priority map, such that only items above that quitting threshold will be inspected prior to executing a target absent response. To the extent that guidance factors impact how many items in a display reach that threshold (by mutual inhibition), there can be substantial shifts in target absent RTs, even with a fixed threshold. In a situation where there is good guidance, only a few items will need to be inspected prior to executing a target absent response. Thus, it might be that there is no real need for one to adaptively set a quitting threshold – the process that might allow for adaptive search times may be how guidance factors influence how many items reach a set quitting threshold.

However, both our data here and that of others (Chun & Wolfe, 1996; Wolfe & van Wert, 2010) suggest that quitting thresholds can be adaptively set and thus may not be fixed. In addition, those same guidance factors which may decrease the number of items that one needs to inspect will also influence target present search times – the better the guidance the faster the search. Thus, the finding that our observers are sensitive to the average search times provides for the possibility that as guidance influences the average target present search times, it may also allow for the fine-tuning of an adjustable quitting threshold. Future work will be needed to determine whether average search time influences quitting thresholds in situations with substantial guidance.

## Conclusion

Using an eye-movement contingent change that inserted targets near fixation at a prespecified time allowed us to manipulate target present search times without changing other factors in the display. Doing so allowed us to demonstrate that people are sensitive to the average amount of time required to find a target and that this information adaptively adjusts a quitting threshold, thereby influencing how completely people search prior to making target absent responses. We also have evidence that mean target present detection times may also influence item-by-item decision criteria, although this was only evident when targets were frequent. We were also able to show that, while the mean detection time has an influence, observers were relatively insensitive to changes in the variability of search times around that mean. These findings provide for a mechanism that allows for flexibly setting quitting thresholds in search tasks that do not have immediate feedback, which may be important given that real-world searches often do not provide this type of feedback.

## Data Availability

Datasets for the three main experiments and the Control Experiment are publicly available on the Open Science Framework at https://osf.io/d7f5e.

## References

[CR1] Awh E, Belopolsky AV, Theeuwes J (2012). Top-down versus bottom-up attentional control: A failed theoretical dichotomy. Trends in Cognitive Sciences.

[CR2] Chun MM, Wolfe JM (1996). Just say no: How are visual searches terminated when there is no target present?. Cognitive Psychology.

[CR3] Faul F, Erdfelder E, Lang A-G, Buchner A (2007). G*Power 3: A flexible statistical power analysis program for the social, behavioral, and biomedical sciences. Behavior Research Methods.

[CR4] Godwin HJ, Menneer T, Riggs CA, Cave KR, Donnelly N (2014). Perceptual failures in the selection and identification of low-prevalence targets in relative prevalence visual search. Attention, Perception, and Psychophysics.

[CR5] Henderson JM (1992). Visual attention and eye movement control during reading and picture viewing. *Eye movements and visual cognition*.

[CR6] Henderson JM, Hollingworth A (2003). Global transsaccadic change blindness during scene perception. Psychological Science.

[CR7] Itti L, Koch C, Niebur E (1998). A model of saliency-based visual attention for rapid scene analysis. IEEE Transactions on Pattern Analysis and Machine Intelligence.

[CR8] Lyu W, Levari DE, Nartker MS, Little DS, Wolfe JM (2020). Prevalence effects on perceptual decisions: Category broadening, elevated miss rates, or both?. Journal of Vision.

[CR9] McConkie GW, Zola D (1979). Is visual information integrated across successive fixations in reading?. Perception & Psychophysics.

[CR10] Mitroff SR, Biggs AT (2014). The Ultra-Rare-Item Effect: Visual Search for Exceedingly Rare Items Is Highly Susceptible to Error. Psychological Science.

[CR11] Pashler H (1987). Detecting conjunctions of color and form: Reassessing the serial search hypothesis. Perception & Psychophysics.

[CR12] Peltier C, Becker MW (2016). Decision processes in visual search as a function of target prevalence. Journal of Experimental Psychology: Human Perception and Performance.

[CR13] Peltier C, Becker MW (2017). Individual differences predict low prevalence visual search performance. Cognitive Research: Principles and Implications.

[CR14] Snodgrass JG (1972). Reaction times for comparisons of successively presented visual patterns: Evidence for serial self-terminating search. Perception & Psychophysics.

[CR15] Torralba A, Oliva A, Castelhano MS, Henderson JM (2006). Contextual guidance of eye movements and attention in real-world scenes: The role of global features in object search. Psychological Review.

[CR16] Treisman AM, Gelade G (1980). A feature-integration theory of attention. Cognitive Psychology.

[CR17] Van Zandt, T., & Townsend, J. T. (1993). Self-terminating versus exhaustive processes in rapid visual and memory search: An evaluative review. *Perception & Psychophysics,**53*(5), 563–580. 10.3758/BF03205204 10.3758/bf03205204 8332425

[CR18] Wolfe JM (1994). Guided Search 2.0 A revised model of visual search. Psychonomic Bulletin & Review.

[CR19] Wolfe JM (1998). What can 1 million trials tell us about visual search?. Psychological Science.

[CR20] Wolfe JM (2021). Guided Search 6.0: An updated model of visual search. *Psychonomic Bulletin and Review*.

[CR21] Wolfe JM, van Wert MJ (2010). Varying target prevalence reveals two dissociable decision criteria in visual search. Current Biology.

[CR22] Wolfe JM, Horowitz TS, van Wert MJ, Kenner NM, Place SS, Kibbi N (2007). Low target prevalence is a stubborn source of errors in visual search tasks. Journal of Experimental Psychology: General.

